# Periodontitis Exacerbates and Promotes the Progression of Chronic Kidney Disease Through Oral Flora, Cytokines, and Oxidative Stress

**DOI:** 10.3389/fmicb.2021.656372

**Published:** 2021-06-11

**Authors:** Ling Li, Ya-Li Zhang, Xing-Yu Liu, Xiang Meng, Rong-Quan Zhao, Lin-Lin Ou, Bao-Zhu Li, Tian Xing

**Affiliations:** ^1^School of Stomatology, Anhui Medical University, Hefei, China; ^2^Key Laboratory of Oral Diseases Research of Anhui Province, College and Hospital of Stomatology, Anhui Medical University, Hefei, China; ^3^Department of Epidemiology and Biostatistics, School of Public Health, Anhui Medical University, Hefei, China

**Keywords:** chronic kidney disease, oral flora, oxidative stress, periodontitis, proinflammatory mediators

## Abstract

Periodontitis is a type of systemic immune inflammation that is caused by the complex infection of a variety of microorganisms in the subgingival plaque and the imbalance of the microbial ecological environment in the mouth. Periodontitis and chronic kidney disease (CKD) share many risk factors, such as obesity, smoking, and age. A growing body of data supports a strong correlation between periodontitis and kidney disease. Evidence supports the role of periodontal inflammation and elevated serum inflammatory mediators in renal atherosclerosis, renal deterioration, and end-stage renal disease (ESRD) development. Periodontitis is a risk factor for kidney disease. However, to our knowledge, there are few studies detailing the possible link between periodontitis and CKD. This review summarizes the possible mechanisms underlying periodontitis and CKD. More importantly, it highlights novel and potential pathogenic factors for CKD, including bacteria, pro-inflammatory mediators and oxidative stress. However, most research on the relationship between periodontitis and systemic disease has not determined causality, and these diseases are largely linked by bidirectional associations. Future research will focus on exploring these links to contribute to new treatments for CKD.

## Introduction

Chronic kidney disease (CKD) is defined by abnormalities in kidney structure or function and is characterized by permanent nephron loss and an eventual decline in the glomerular filtration rate (GFR) (Zalups and Diamond, 1987). CKD can be classified into glomerular, vascular, renal tubulointerstitial, cystic, and other congenital diseases. Treatment options include dialysis, mainly peritoneal and hemodialysis (HD), or kidney transplantation (Eckardt et al., 2009). Over the last 10 years, both the prevalence and incidence of CKD have constantly increased, NS Uts prevalence is greater than 10% in many countries (Eckardt et al., 2013). Furthermore, CKD accounts for 1.1 million deaths worldwide according to the Global Burden of Disease study. Overall, due to its poor prognosis, the death rate from CKD has increased by 31.7% over the past 10 years, making it one of the fastest growing leading causes of death, along with diabetes and dementia. In the same study, CKD ranked 17th in the number of years of death globally, with an 18.4% increase since 2005 and the third largest increase among all leading causes of death. The high prevalence, rapid progression and poor prognosis of CKD mean that it is necessary to find new methods to prevent and control CKD development (GBD 2015 Mortality and Causes of Death Collaborators, 2016; Neuen et al., 2017).

Periodontitis is a complex infection caused by many microorganisms in the subgingival plaque. It is a chronic infectious disease caused by an ecological imbalance of microorganisms in the oral cavity. When a patient has periodontitis, the supporting structures of the teeth, including the periodontal membrane and alveolar bone, are destroyed (Pihlstrom et al., 2005). Approximately 10% of the world’s population has severe periodontitis (Frencken et al., 2017). In addition, periodontitis may have a negative impact on body balance control and the development of persistent disease (Brito et al., 2012). Therefore, periodontitis is a highly prevalent chronic inflammatory disease with negative and far-reaching effects on many aspects of daily life (O’Dowd et al., 2010).

There have been new discoveries on the relationship between periodontitis and systemic disease. By investigating the pathogenic mechanisms of periodontal disease (PD), it was found that PD is linked to diseases outside the oral cavity, such as atherosclerosis, diabetes, stroke, and coronary heart disease (Preshaw et al., 2012; Beck et al., 2018; Gualtero et al., 2018; Sen et al., 2018). The relationship between PD and kidney disease has also been discussed (Kshirsagar et al., 2009; Franca et al., 2017). Recently, a strong association between PD and CKD has been highlighted in the literature (Kshirsagar et al., 2005). Although the pathogenesis of CKD and PD has not been fully elucidated, the relationship between these two diseases will be explored in this review. Here, we specifically discuss the various potential etiologies of these conditions from a broad perspective. Furthermore, we sought to determine whether CKD is a dysregulated inflammatory response or the effect of a specific pathogen effect resulting from the seeding of organisms from oral PD and chronic infections.

## Impact of PD on the Pathogenesis of CKD

### Oral Flora

Microbial complexes in the subgingival biofilm were classified into five groups: red, green, orange, yellow, and purple. In particular, the red group, which is composed of *Tannerella forsythia*, *Treponema denticola*, and *Porphyromonas gingivalis*, has been identified as one of the main causes of PD (Socransky et al., 1998). The detection rate of some oral bacteria in CKD patients is higher than that in healthy individuals. A study showed an increased frequency of *P. gingivalis* (*P* = 0.008), *T. forsythia* (*P* = 0.013), and *T. denticola* in CKD patients (Bastos et al., 2011). Another study showed that 9% of American adults ≥40 years of age had CKD; of these patients, 22% had a high *Aggregatibacter actinomycetemcomitans* antibody titer, 24% had a high *P. gingivalis* antibody titer, 9% had PD, and 17% were edentulous (Fisher et al., 2008). After simultaneously adjusting for recognized risk factors, adults with high *A. actinomycetemcomitans* titers were less likely to have CKD, but adults with edentulism were more likely to develop CKD (Fisher et al., 2008). Specific microbes enriched in the subgingival flora of peritoneal dialysis patients with periodontitis (Zhang et al., 2020). Therefore, this review mainly discusses the relationship between these oral bacteria and CKD.

**FIGURE 1:**
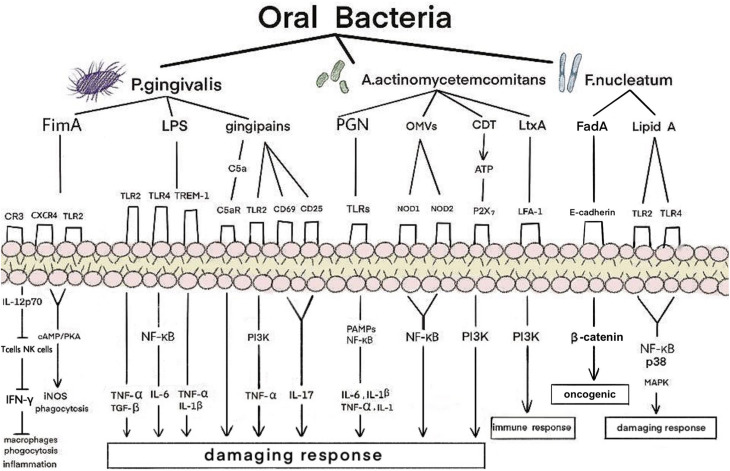
Mechanism of oral bacteria on kidney in patients with periodontitis. *P. gingivalis*, *A. actinomycetemcomitans*, and *F. nucleatum* have adverse effects on the kidneys. *P. gingivalis* produces an inflammatory response mainly through the combination of fimbriae proteins, LPS and gingipains with corresponding receptors. *A. actinomycetemcomitans* bind to the corresponding receptors through PGN, OMVs, CDT, and LtxA, and generate damaging and immune responses through the PAMPs, NF-κB, and PI3K signaling pathway. *F. nucleatum* bind to the corresponding receptors through FadA and lipidA and produce oncogenic and damaging response through the NF-κB, p38, and MAPK signaling pathway. LPS, lipopolysaccharide; OMVs, outer membrane vesicles; CDT, cytolethal distending toxin; LtxA, leukotoxin; CXCR4, CXC-chemokine receptor 4; TREM-1, trigger receptor on myeloid cells 1; LFA-1, lymphocyte function-associated antigen 1; CR, complement receptor; TLR, toll-like receptor; iNOS, inducible NO synthase; cAMP, cyclic adenosine monophosphate; PKA, protein kinase A; NF-κB, nuclear factor kappa B; TNF-α, tumor necrosis factor-α; TGF-β, transforming growth factor-β; IL, interleukin; PAMPs, pathogen-associated molecular patterns; PI3K, phosphatidylinositol-4,5-bisphosphate 3-kinase; FadA, Fusobacterium adhesion; MAPK, mitogen-activated protein kinases.

Oral bacteria can spread by circulating blood and swallowing, and they induce inflammatory responses in distant tissues via dissemination using the circulatory system (Chou et al., 2005; Bastos et al., 2011; Louren*ς*o et al., 2018). *P. gingivalis* and *T. forsythia* have an average of 10^6^–10^8^ copies per mL in subgingival and salivary samples in periodontitis patients (Saygun et al., 2011). In patients with periodontitis, 10^8^–10^10^ copies of the keystone periodontal pathogen *P. gingivalis* can be swallowed each day (Saygun et al., 2011; von Troil-Lindén et al., 2016). Oral bacteria can enter the circulation and cause bacteremia by actively crossing the periodontal epithelium (Takeuchi et al., 2011; Tomas et al., 2012; Reyes et al., 2013). While direct cellular invasion of renal tissue by periodontal pathogens has not been detected in periodontitis, some oral bacteria reportedly invade coronary and aortic endothelial cells (Chou et al., 2005). *A. actinomycetemcomitans*, *T. denticola* and *P. gingivalis* are capable of invading endothelial cells (Schenkein et al., 2000; Dorn et al., 2002; Takahashi et al., 2006) and have been detected in atherosclerotic plaques, heart valves, aortic aneurysms, carotid arteries, and coronary vessels (Marques, da Silva et al., 2005; Nakano et al., 2009). In the hearts and aortas of toll-like receptor (TLR)2^–/–^TLR4^–/–^ deficient mice, *P. gingivalis*, *T. denticola*, and *Fusobacterium nucleatum* genomic DNA were positive in all organs, indicating their hematogenous dissemination based on the highest genomic DNA presence (Chukkapalli et al., 2018). The direct or indirect effects of circulating bacteria, inflammatory mediators and/or immune complexes from infected or inflamed periodontal tissues on other body sites are some of the main mechanisms that contribute to systemic inflammation (Li et al., 2000). Strong evidence has accumulated to indicate that the pathogenic microbiota and chronic inflammation established in periodontitis contribute to the progression of CKD (Shultis et al., 2007) (Figure 1).

#### P. gingivalis

Red complex bacteria have been demonstrated to be the predominant microbial species involved in PD progression; among them, *P. gingivalis* is regarded as a keystone pathogen in adult periodontitis (Hajishengallis et al., 2012; Maekawa et al., 2014). It is a common bacterium in the oral microbiome and a successful colonizer of oral epithelial cells (Lamont et al., 2018). The virulence factors of *P. gingivalis*, including fimbriae proteins, lipopolysaccharide (LPS) and gingipains, play important roles in pathogen interactions and inflammation. *P. gingivalis* fimbriae (FimA), a filamentous structure identified on the surfaces of these bacteria, play a role in the invasion and colonization of host tissues (Dashper et al., 2017). *P. gingivalis* FimA is also closely associated with bacterial adhesion and biofilm formation (Dashper et al., 2017); they may affect the kidney by activating adhesion signals, phagocytosis, inflammatory response, and inducible nitric oxide synthase (iNOS). Inflammation that fails to clear the pathogen can be characterized as non-productive when the infection becomes chronic. With these two chronic diseases, long-term exposure to various byproducts of the disease and complications related to disruptions in homeostasis can lead to distal effects in a variety of body functions and sites.

*P. gingivalis* activates adhesion signals by stimulating macrophages and neutrophils. Complement receptor 3 (CR3) can use this pathway to enhance the interaction between FimA and CR3 on the cell surface. Activated CR3 interacts with gingival flora to reduce IL-12p70. IL-12p70 production by macrophages activates T cells and NK cells to produce IFN-γ, which in turn activates the bactericidal function of macrophages (Trinchieri, 2003). Therefore, CR3 blockade may represent a promising immunomodulatory approach for controlling human periodontitis and associated systemic diseases (Cai et al., 2019). *P. gingivalis* FimA can activate the interaction of CXC-chemokine receptor 4 (CXCR4) and TLR2 in macrophages and induce cyclic adenosine monophosphate (cAMP)-dependent protein kinase A (PKA) signaling. However, they can inactivate glycogen synthase kinase-3β (GSK3β) and disrupt iNOS-dependent phagocytosis of macrophages. *P. gingivalis* FimA activate TLR2/TLR1 on macrophages and induce the production of a small amount of cAMP. Moreover, the continuous increase in cAMP activates PKA in macrophages and destroys the bactericidal function of iNOS, which is dependent on NF-κB (Wang et al., 2010). This indicates that phagocytosis by macrophages may induce kidney injury. In addition, the elevation of cAMP leads to a decrease in the production of nitric oxide (NO) by renal endothelial cells. The lack of NO can lead to severe renal fibrosis and enhance the interaction between neutrophils and endothelial cells (Herrera et al., 2011). The regulatory action of the cAMP/PKA pathway on cell activity depends mainly on the binding of phosphorylated cAMP response element binding protein (CREB) to the nuclear coactivator CREB binding protein (CBP) (Dyson and Wright, 2016). Therefore, FimA may be one of the factors affecting the kidneys.

#### Lipopolysaccharide (LPS)

Lipopolysaccharide is a component of the cell wall of Gram-negative bacteria and plays an important role in the activation of the inflammatory response in chronic periodontitis (Golz et al., 2014; Holden et al., 2014). *P. gingivalis* LPS may induce diabetic renal inflammation, such as glomerulosclerosis and tubulitis with infiltration of Mac-1/podoplanin-positive macrophages, via glomerular overexpression of vascular cell adhesion molecule-1 (VCAM-1) and E-selectin (Kajiwara et al., 2021). In one study, mice were intraperitoneally injected with *P. gingivalis* LPS at a concentration of 5 mg/kg every 3 days for 1 month. Microarray analysis showed that the 10 genes with the highest expression levels in the kidney were stimulated with *P. gingivalis* LPS. Among them, five genes (*Saa3*, *Ticam2*, *Reg3b*, *Ocxt2a*, and *Xcr1*) are known to function. Mouse primary kidney glomerular endothelial cells (KEC) were cultured with or without *P. gingivalis* LPS. The expression levels of Saa3, Ticam2, Reg3b, Oxct21, and Xcr1 significantly increased in the 1000 ng/mL *P. gingivalis* LPS groups compared with the controls. when compared with the controls. In addition, upregulation of the expression levels of *Saa3*, *Ticam2*, *Reg3b*, *Ocxt2a*, and *Xcr1* may be related to CKD pathogenesis. The five overexpressed genes in the kidney-derived endothelial cells were induced by LPS (Harada et al., 2018). Each of these five genes has its own role. *Saa3* expression levels have been utilized to ascertain the number of infiltrated macrophages during chronic inflammation in obese adipose tissue (Sanada et al., 2016). Mal/Toll/IL-1R (TIR) domain-containing adaptor molecule 2 (*Ticam2*), also known as TRAM (TRIF-related adaptor molecule), acts as a sorting adaptor on the early endosomes for the recruitment of TIR-containing adapter molecule (TICAM)-1 to TLR4, which is known to recognize LPS (Klein et al., 2015). *Reg3b* regulates important biological processes such as immune responses, cell adhesion, and protection via apoptosis (Ferreira et al., 2011). Increased *Oxct2* expression may be related to diabetic CKD (Harada et al., 2018). Both dendritic and endothelial cells may contribute to the overexpression of *Xcr1* in the kidneys (Lei and Takahama, 2012). These findings demonstrate that *P. gingivalis* LPS may have an effect on the kidneys.

*P. gingivalis* LPS can also affect the kidneys through M1 and M2 macrophages. One study indicated that *P. gingivalis* LPS activated M1 and M2 macrophages mainly via TLR2; this was accompanied by the observation that high concentrations of LPS produced NO by stimulating M1 macrophages, while low concentrations primarily increased cytokine expression (Holden et al., 2014). Another study showed that LPS administration promoted urinary protein production, accumulation of type I collagen in the glomeruli, and increased interleukin-6 (IL-6), tumor necrosis factor-α (TNF-α), and transforming growth factor-β (TGF-β) expression in the renal cortex of the glomeruli of diabetic mice (Chou et al., 2005). It is believed that *P. gingivalis* LPS is involved in inducing the production of proinflammatory cytokines such as TNF-α and IL-6 in glomerular endothelial cells by binding to TLR2 and TLR4, as well as the production of anti - inflammatory cytokines such as TGF-β to promote glomerular sclerosis (Sawa et al., 2014). The initial inflammatory response of macrophages to *P. gingivalis* was mainly dependent on myeloid differentiation primary response 88 (MyD88), which requires synergistic TLR2 and TLR4 signaling. Although both TLR2 and TLR4 are involved in TNF-α production in macrophages, *P. gingivalis* preferentially exploits TLR2 in endotoxin-tolerant bone marrow-derived macrophages (BMDMs) to trigger excessive TNF-α production (Cai et al., 2019). *P. gingivalis* FimA inhibit the ability to stimulate IL-8 production in endothelial cells, leading to local chemokine paralysis and an uncontrolled immune response, resulting in renal dysfunction (Cai et al., 2019). Thus, *P. gingivalis* LPS induces the expression of TLR4 and inflammatory cytokines and activates the NF-κB signaling pathway (Kim et al., 2018).

*P. gingivalis* LPS also upregulates the expression and release (shedding) of the surface-bound trigger receptor on myeloid cells 1 (TREM-1) into a soluble form (sTREM-1). TREM-1 is a cell surface receptor expressed in innate immune cells. TREM-1 has synergistic effects with cell surface pattern recognition receptors (PRRs) such as toll-like- and NOD-like- receptors to accelerate the production of proinflammatory cytokines such as TNF-α and IL-1β. Peptidoglycan recognition protein 1 (PGLYRP1) is a secreted antibacterial protein that acts against Gram-positive and -negative bacteria. The components of periodontal bacteria do not bind to TREM-1 directly, but instead bind the PGLYRP1 ligand. In periodontitis patients with renal diseases, the correlation between sTREM-1 and PGLYRP1 has been shown to be positively correlated with PGLYRP1 and proinflammatory cytokines in patients with CKD before dialysis (Bostanci et al., 2013; Nylund et al., 2018).

#### Gingipains

Some virulence factors are known to be involved in the pathogenicity of *P. gingivalis*, including fimbriae and LPS; however, the major virulence factor is a family of cysteine proteases called gingipains. Gingipains are proteinases that are secreted by *P. gingivalis* and are involved in a variety of functions, including the acquisition of essential nutrients, invasion of host tissues, inactivation of cytokines and their receptors, and attenuation of neutrophil antibacterial activities. Thus, they are necessary for the survival of *P. gingivalis* in the anaerobic host environment (Guo et al., 2010; Bostanci and Belibasakis, 2012). Gingipains cause kidney damage by activating an inflammatory response in various immune cells, including neutrophils, macrophages, and T cells.

*In vitro* experiments have shown that gingipains of *P. gingivalis* stimulate TLR2-phosphatidylinositol-4,5-bisphosphate 3-kinase (PI3K) signaling. They then stimulate activated neutrophils to produce the proinflammatory cytokine TNF-α, thereby prolonging the maturation and survival time of host cells (Makkawi et al., 2017). *P. gingivalis*-specific gingipains can decompose the fifth complement component (C5) into C5a (fragment of complement protein C5) and C5b in two ways: directly exerting the transforming activity of C5, and activating thrombin to activate prothrombin to replace the C5 transforming enzyme (Hajishengallis et al., 2013). More importantly, recent studies have discovered an important mechanism of combined action between the C5a receptor (C5aR) and TLR2 in macrophages to promote the adaptability of *P. gingivalis* (Wang et al., 2010). Gingipains can directly induce the expression of CD69 and CD25 on T cells and induce IL-17 production (Yun et al., 2007). Taken together, these studies demonstrate that gingipains can adversely affect the kidneys.

#### A. actinomycetemcomitans

*A. actinomycetemcomitans* is an exogenous oral bacterium associated with aggressive forms of periodontitis. This organism possesses a large number of virulence factors with a wide range of activities that enable it to colonize the oral cavity, invade periodontal tissues, evade host defenses, initiate connective tissue destruction, and interfere with tissue repair (Raja et al., 2014). More recently, renal lesions caused by other pathogens were found to be positive for the same molecular marker. A study reported the case of a 64-year-old man who experienced repeated fever for several months and presented with progressively deteriorating renal function. He had previously undergone aortic valve replacement. *A. actinomycetemcomitans*, a component of the oral flora, was found in the blood culture of this patient. Renal biopsy revealed diffuse proliferative glomerulonephritis (Komaru et al., 2018). This is the first report of nephritis-associated plasmin receptor (NAPlr) positivity in the glomeruli after systemic infection with *A. actinomycetemcomitans*. *A. actinomycetemcomitans* acts primarily on the kidney through PGN, outer membrane vesicles (OMVs), cytolethal distending toxin (CDT), and leukotoxin (LtxA).

Similar to LPS, the bacterial wall of oral bacteria (PGN) can stimulate blood cells to produce cytokines. PGN-associated lipoproteins can activate macrophages to produce IL-6, IL-1β, and TNF-α by binding to TLR2 receptors (Ihalin et al., 2018). LPS and PGN are prototypical classes of pathogen-associated molecular patterns (PAMPs) that are recognized by TLRs, leading to the activation of proinflammatory signaling pathways (Kawai and Akira, 2011). Together with IL-1, TNF-α activates NF-κB, which promotes the expression of apoptotic genes in nephritis and activates cell proliferation and differentiation, as well as permanent cell damage in the kidneys.

*A. actinomycetemcomitans* can extend their pathogenicity by releasing membrane vesicles (MVs), which represent a very basic and relevant mode of protein export from bacteria that has been referred to as ‘Type Zero’ secretion (Rompikuntal et al., 2015). There is evidence supporting that OMV internalization is a prerequisite for the induction of a proinflammatory response (Bielig et al., 2011). Moreover, colocalization analysis revealed that internalized OMVs colocalized with the endoplasmic reticulum and carried antigens, as detected using an antibody specific to whole *A. actinomycetemcomitans* serotype A cells. Consistent with the fact that the internalization of *A. actinomycetemcomitans* OMVs mediates intracellular antigen exposure, the vesicles acted as strong inducers of the cytoplasmic PGN sensor NOD1- and NOD2-dependent NF-κB activation (Thay et al., 2014). Thus, *A. actinomycetemcomitans* OMVs may cause a proinflammatory response in the kidneys.

*A. actinomycetemcomitans* is the only oral species that is known to express CDT. An estimated 66–86% of its strains express CDT, and its presence has been associated with the occurrence of PD (Fais et al., 2016). Cells of the immune system are also highly susceptible to the cell cycle arrest and apoptotic action caused by CDT, as has been demonstrated in human T cells (Shenker et al., 1999), B cells (Sato et al., 2002), and mononuclear cells (Akifusa et al., 2001). *A. actinomycetemcomitans* produces an immunosuppressive factor (ISF) capable of impairing human lymphocyte function by perturbing cell cycle progression. ISF is the product of the CDTB gene, one of the three genes encoding the CDT family (Shenker et al., 1999). CDT from *A. actinomycetemcomitans* induces cell distension and G2 cell cycle arrest in HeLa and B cell hybridoma cells. CDT-induced p21^ CIP1/WAF1^ promotes G2 cell cycle arrest in several types of mammalian cells, such as B-lineage cells and fibroblast-like cells. *A. actinomycetemcomitans* has been associated with periodontitis and systemic infections in humans (Sato et al., 2002). An experimental protocol was used to determine whether recombinant CDT proteins were able to induce cytokine production in human PBMCs. Individual CDT proteins are able to induce IL-1β, IL-6, and IL-8 synthesis by PBMCs (Bielig et al., 2011); furthermore, CDT-mediated increases in extracellular ATP and ATP-induced P2X_7_ activation likely contribute to NOD-like receptor family pyrin domain containing 3 (NLRP3) assembly and activation. Furthermore, treating lymphocytes with CDT leads to perturbations in PI3K signaling, including phosphatidylinositol-3,4,5-triphosphate (PIP3) depletion, and decreases both Akt and GSK3β phosphorylation. In addition, CDT treatment decreases kinase activity in Akt but increases GSK3β. *A. actinomycetemcomitans* CDT activates the NLRP3 inflammasome in human macrophages, leading to the release of proinflammatory cytokines (Shenker et al., 2015). Therefore, we infer that *A. actinomycetemcomitans* may affect the kidneys by acting on immune cells.

LtxA is an important protein toxin of *A. actinomycetemcomitans.* LtxA affects the kidney mainly by acting on white blood cells to produce an immune response. Lymphocyte function-associated antigen 1 (LFA-1) is expressed in all white blood cells (Miller et al., 1986), and LtxA of *A. actinomycetemcomitans* affects different leukocyte populations (Johansson, 2011). A region of LtxA contains a series of 14 tandemly repeated amino acid sequences in the repeat region of the toxin and is shown to be responsible for receptor binding to LFA-1 (Hirschfeld et al., 2016). LtxA activates neutrophil degranulation, causing a massive release of lysosomal enzymes, net-like structures, and matrix metalloproteinases (MMPs) and induces apoptosis in lymphocytes (Hirschfeld et al., 2016). However, pretreatment of cells with a PI3K inhibitor significantly inhibited LtxA-mediated killing (DiFranco et al., 2012), suggesting that LtxA of *A. actinomycetemcomitans* can affect the kidneys through the PI3K pathway.

#### Other Bacteria

In addition to *P. gingivalis* and *A. actinomycetemcomitans*, oral microbiota may adversely affect the kidneys. *F. nucleatum* includes five subspecies: *polymorphum*, *nucleatum*, *vincentii*, *fusiforme*, and *animalis* (Dzink et al., 1990). In a previous study, *P. gingivalis*, *T. denticola*, *T. forsythia*, and *F. nucleatum* were associated with innate immune signaling and induction of periodontitis and atherosclerosis in a mouse model (Chukkapalli et al., 2018). An important factor in *T. forsythia* virulence is the serine proteinase inhibitor (serpin) protein, which is known as miropin. This protein inhibits serine proteases from neutrophils, thus protecting the bacteria against the proteolytic effects of neutrophils (Ksiazek et al., 2015). This suggests that these oral bacteria may elicit immune responses to the body, which may affect the kidneys. Atherosclerotic plaque progression was markedly reduced in infected TLR2^–/–^TLR4^–/–^ mice or heterozygous mice, indicating a profound effect on plaque growth. However, bacterial genomic DNA was detected in multiple organs in TLR2^–/–^TLR4^–/–^ mice, indicating intravascular dissemination from gingival tissue to the heart, aorta, kidney, and lungs. In addition, *P. gingivalis*, *T. denticola*, and *F. nucleatum* genomic DNA were also positive in the kidney, but the bacteria were not identified (Chukkapalli et al., 2018). These studies show that some oral bacteria can adversely affect the kidneys.

Among these bacteria, *F. nucleatum* is the oral pathogen that is most commonly found at sites of systemic infection. In a previous study, ApoE^null^ mice (*n* = 23) were orally infected with *F. nucleatum* subsp. *vincentii ATCC* 49256. The mice developed bacteremia symptoms, with *F. vincentii* genomic DNA detected in systemic organs (heart, aorta, kidney, liver, and lung) (Velsko et al., 2015). *F. nucleatum* lipid A is a hexa-acylated fatty acid that is composed of tetradecanoate (C14) and hexadecanoate (C16) and is structurally similar to *Escherichia coli* lipid A. This structural similarity may explain the strong activity of *F. nucleatum* via TLR4 (Asai et al., 2007). Furthermore, the same study showed that TLR2/TLR4 and MyD88 are required for the best activation of NF-κB and mitogen-activated protein kinases [MAPKs, including p38, extracellular signal-regulated kinase (ERK), and Jun N-terminal protein kinase (JNK)] in response to *F. nucleatum* (Park et al., 2014). In gingival epithelial cells (GECs), *F. nucleatum* infection was sufficient to induce caspase-1 activation in an NLRP3-dependent manner and the secretion of the danger signals ASC and high-mobility group box 1 protein (HMGB1) (Bui et al., 2016). Caspases are endoproteases that cleave peptide bonds in a cysteine-dependent and -directed manner. Caspases can cause cell death (pyroptosis) and inflammation (McIlwain et al., 2015). The active invader species *F. nucleatum* can independently invade host cells in part using extracellular adhesin and invasion molecules, such as Fusobacterium adhesion (FadA) (Xu et al., 2007; Ikegami et al., 2009). It has been reported that *F. nucleatum* FadA binds to E-cadherin, thus activating β-catenin signaling and differentially regulating inflammatory and oncogenic responses (Rubinstein et al., 2013). Moreover, significantly redundant FadA paralogs could contribute to severe mucosal inflammation (Sekizuka et al., 2017). Taken together, cell death and inflammation also play important roles in the kidneys.

### Impact of Proinflammatory Mediators on Progression of CKD

#### Cytokines

**FIGURE 2:**
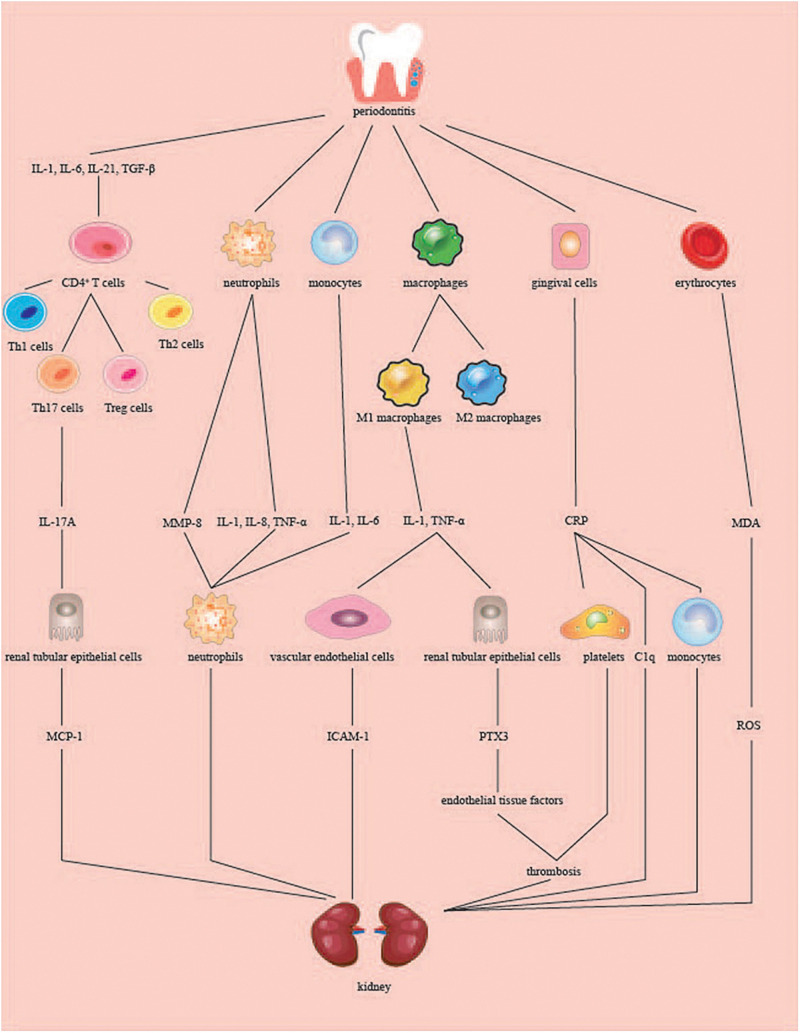
Role of periodontitis in kidney inflammation. When periodontitis occurs, periodontal tissues produce inflammatory cytokines, which are translocated to the kidneys through the blood circulation. CD4^+^ T cells are today classified into four major subsets: Th1, Th2, Th17, and Treg cells. IL-1, IL-6, IL-21, TGF-β induced CD4(+) T cells to differentiate into Th17A cells, and IL-17A produced stimulated renal tubular epithelial cells to produce MCP-1. The neutrophils and monocytes induced by periodontitis produced MMP-8, IL-1, IL-6, IL-8, and TNF-α, among which MMP-8, IL-8, and IL-6 acted on the neutrophils at the site of inflammation and produced an inflammatory response. IL-1 and TNF-α stimulated by macrophages can promote the production of ICAM-1 by vascular endothelial cells on the kidney or promote the production of PTX3 by renal tubular epithelial cells. PTX3 increases endothelial tissue factors involved in renal thrombosis. CRP produced by gingival cells stimulates platelet aggregation to form thrombus and combine C1q and factors of complement. Periodontitis raises the level of MDA in red blood cells, plasma and local tissues, which leads to the upregulation of ROS. Th, T-helper; Treg cells, regulatory T cells; MCP-1, monocyte chemotactic protein-1; MMP-8, matrix metalloproteinase 8; TNF-α, tumor necrosis factor alpha; ICAM-1, intercellular adhesion molecule-1; PTX3, pentraxins 3; CRP, C-reactive protein; MDA, malondialdehyde; ROS, reactive oxygen species.

Cytokines such as IL-1, IL-6, IL-8, IL-17, and TNF-α are associated with periodontitis and kidney disease (Figure 2). The IL-1 gene encodes inflammatory mediators involved in the pathogenesis of periodontitis and CKD (Braosi et al., 2012). In patients with CKD, it was found that plasma concentrations of IL-6 and TNF-α appeared to be more sensitive markers of odontogenic inflammation in CKD patients (Niedzielska et al., 2014). In another study, there was a positive correlation between clinical indicators and TNF-α and IL-8 levels in gingival crevicular fluid (GCF) extracted from HD patients. The levels of TNF-α and IL-8 in the GCF of HD patients were significantly higher than those in the healthy group (Dag et al., 2010).

The production of IL-1β, IL-8, and TNF-α can be induced by stimulating neutrophils in the blood through the LPS of periodontal pathogens (Yoshimura et al., 1997). IL-1 and IL-6 can be produced by stimulating peripheral blood mononuclear cells through pathogenic bacteria that express CDT (Yoshimura et al., 1997). According to a previous study, the soluble delivery of active TNF leptin (LEP) in macrophages can be achieved through the role of pathogenic proteases (Bryzek et al., 2014). IL-6, TGF-β, IL-1, and IL-21 induce the differentiation of mouse and human CD4(+) T cells into T-helper 17 (Th17) cells (Turner et al., 2010). Chronic inflammation in periodontal tissues stimulates Th17 cells to produce IL-17 (Moutsopoulos et al., 2012). IL-17 can enter the serum of patients, and studies have shown that the serum IL-17 levels of the patients were higher than those of the healthy group (Schenkein et al., 2010). Based on the above studies, we hypothesized that the pathogenic oral bacteria’s established by the periodontitis mentioned above might cause the release of cytokines by immune cells in the blood, thereby causing kidney damage.

IL-6 further increases renal inflammation by recruiting more neutrophils into the damaged kidneys and aggravates acute renal failure in ischemic mice (Kielar et al., 2005). IL-8 is a harmful chemokine involved in renal damage in glomerulonephritis and is generally associated with neutrophil accumulation and neutrophil-dependent edema at sites of acute and chronic inflammation (Niemir et al., 2004). TNF-α is involved in the progression of renal insufficiency through its cytotoxic and proinflammatory effects, such as by inducing endothelial cells (ECs) to express intercellular adhesion molecule-1 (ICAM)-1 (Izawa-Ishizawa et al., 2012). Chronically elevated circulating IL-17A levels can activate the immune response and damage the kidney tissue. IL-17A promotes upregulation of genes related to proinflammatory factors, such as IL-23 mRNA. IL-17A also increases the production of monocyte chemotactic protein-1 (MCP-1) in renal tubular epithelial cells. MCP-1 is a key driver of renal inflammatory cell aggregation and has been proposed as a biomarker for kidney damage (Orejudo et al., 2019). IL-17A can aggravate kidney inflammatory diseases such as diabetic nephropathy, hypertension, and lupus nephritis (Lavoz et al., 2019; Orejudo et al., 2019; Zhang and Li, 2020). DNA was obtained from oral mucosal cells, and IL1AC889T, IL1BC511T, IL1BC3954T, and IL1RN (intron 2) polymorphisms were analyzed by PCR-RFLP (Braosi et al., 2012). It was found that the IL1RN^∗^1 allele was associated with an almost fourfold increase in risk of developing CKD. The IL1RN^∗^2 allele was associated with a threefold increase in risk of PD in CKD patients. CKD in PD patients was associated with allele T for polymorphism IL1B + 3954. IL1 may be related to the gene clustering polymorphisms in PD and CKD (Schreiber et al., 2012). Cytokines may be a pathway through which periodontitis causes kidney disease; however, more research is needed.

#### Acute Phase Proteins (APPs)

The levels of various APPs in GCF, such as C-reactive protein (CRP), PTX family protein, fibrin, and haptoglobin, are affected by the local periodontitis response. The increase in APP concentration in plasma promotes the production of proteolytic enzymes in the body, resulting in renal endothelial cell damage, endothelial cell permeability increase, glomerular filtration dysfunction, and ultimately aggravate kidney disease, which is often considered to be an important bridge between periodontitis and systemic inflammation.

#### C-Reactive Protein (CRP)

A study on the effects of non-surgical periodontal treatment on serum CRP levels found that systemic inflammation in patients with CKD resulted from periodontitis (Yazdi et al., 2013). Periodontitis treatment can reduce serum CRP levels in patients with CKD. In 2011, a study found that the average serum CRP levels in 50 periodontitis patients were significantly higher than in healthy controls. Patients with severe periodontitis generally have a high level of clinical attachment loss, and the mean CRP level is higher in patients with moderate periodontitis (Pejcic et al., 2011). CRP can combine C1q and complement factors by activating the classical and alternative complement pathways. It can induce thrombus formation by activating white blood cells, inducing cytokine release and monocyte aggregation, and inducing platelet aggregation. CRP also damages the endothelial cells of the kidney, promotes atherosclerotic plaque formation in arteries, and results in renal artery occlusion, poor blood circulation, and aggravation of kidney disease (Melnikov et al., 2020).

#### Pentraxins (PTXs)

Pentraxins (PTXs) are classical APPs that have been known for over a century. They are typical signs of acute phase response and emerging biomarkers of inflammation that can modulate and play key roles in the immune inflammatory response. The prototype protein of the long-entrain group is pentraxins 3 (PTX3). PTX3, which is associated with CKD and periodontitis, is a truly independent indicator of disease activity. PTX3 amplifies the inflammatory process by binding to the C1q component of the complement cascade (Pradeep et al., 2012). In different cell types, such as endothelial phagocytes, smooth muscle cells, fibroblasts, and glial cells, TNF-α and ILs upregulate PTX3 transcription and are involved in atherogenesis. A 2011 study on the relationship between plasma PTX3 and CKD suggested that neutrophils and monocytes accumulate at the lesion site and that cytokines such as TNF-α, IL-1, and IL-6 are amplified (Pradeep et al., 2012). Furthermore, PTX3 is highly expressed in periodontitis; after experimental induction of periodontitis, serum PTX3 levels were significantly increased 24 h after injection and continued to increase for 21 days (Leira et al., 2020). When periodontal inflammation occurs, lymphocytes and monocytes in the blood are activated and proliferate. Monocytes and T cells release IL-1 and TNF-α in renal tubules and promote PTX3 mRNA expression in renal tubular epithelial cells. Increased PTX3 production can also be jointly activated by IL-1 and CD40L, as shown in renal tubular epithelial cells (Nauta et al., 2005). PTX3 can aggravate kidney disease during inflammation, with (Valente et al., 2019) studies showing that decreased renal function may be associated with higher PTX3 levels in patients with CKD (Sjoberg et al., 2016). PTX3 can increase the expression of endothelial tissue factors and participate in the formation of thrombosis and vascular ischemia of tissue factors by endothelial cells (Napoleone et al., 2002). PTX3 promotes the production of platelet activating factor and proinflammatory mediators in mesangial cells, which can cause platelets to adhere to renal tissue and cause damage (Bussolati et al., 2003).

#### Asymmetric Dimethyl Arginine (ADMA)

Asymmetric dimethyl arginine is used to assess endothelial function and is the most effective endogenous inhibitor of nitric oxide synthase (NOS). NOS is a key regulator of vascular tension and plays a key role in endothelial dysfunction, atherosclerosis progression, and cardiovascular disease (Oliva-Damaso et al., 2019). Endothelial dysfunction is related to periodontitis, and pathogenic bacteria or products of PD can directly affect endothelial function (Gurav, 2014). These pathogens trigger an inflammatory response throughout the body, which can have harmful effects on the blood vessel walls (Gurav, 2014). The presence of NOS and cyclooxygenase (COX) isozymes were measured in mice killed 7 days after unilateral periodontitis induction, and COX and NOS gene expression in the aorta was analyzed by real-time polymerase chain reaction (RT-PCR) (Campi et al., 2016). Consistent with previous findings, the expression of endothelial nitric oxide synthase (eNOS) in mice decreased and the expression of iNOS and COX-2 increased. This suggests that ligative periodontitis causes endothelial dysfunction in mice. Renal endothelial injury may be a pathway for periodontal pathogens that cause other diseases (Grubbs et al., 2017). A study linking CKD with endothelial dysfunction and periodontitis assessed the effect of periodontal therapy on renal function in patients with CKD in the short term. The results showed a significant decrease in ADMA levels after periodontal treatment, which may indicate improved endothelial function and a better prognosis for CKD development (Almeida et al., 2017). Therefore, periodontitis can lead to elevated ADMA levels, resulting in renal endothelial dysfunction.

#### Matrix Metalloproteinases (MMPs)

Matrix metalloproteinases are found in the periodontium and are calcium-dependent zinc-containing proteolytic enzymes that are responsible for organ oogenesis, normal tissue turnover, and tissue breakdown (Nylund et al., 2015). Neutrophils are the main source of matrix metalloproteinase 8 (MMP8). Studies on the content of MMP8 in saliva have suggested that periodontal inflammation triggers systemic diseases, including CKD (Nylund et al., 2015). Periodontal bacteria stimulate immune cells to release MMP8 into the bloodstream, and studies have shown that serum MMP8 levels are higher in patients with generalized and invasive periodontitis (Nizam et al., 2014). Oral bacteria such as *F. nucleatum*, *P. gingivalis* and *T. denticola* can induce the release and activation of MMP8 by neutrophils (Ding et al., 1997). *A. actinomycetemcomitans* leukotoxin reportedly affects different white blood cell populations and activates neutrophils to degranulate, resulting in lysosomal enzymatic reticular structure and matrix proteinase (Ding et al., 1997). MMP family performs various functions in the kidneys. MMPs, which are collectively responsible for maintaining extracellular matrix (ECM) protein scaffolds around the body, are involved in renal fibrosis and chronic remodeling, diabetic nephropathy, polycystic kidney disease, and glomerulonephritis. MMPs may also be involved in the regulation of renal inflammation (Basu et al., 2015). MMP8 and its various molecular subtypes have been found in the urine of diabetic nephropathy patients, and MMP8 reportedly plays an important role in the pathogenesis of diabetic nephropathy. MMP8 may also mediate tissue destruction by lysing collagen and protease inhibitors (serpins) (Nylund et al., 2015). There is a large amount of evidence suggesting that MMPs are associated with both periodontitis and kidney disease; however, the suggestion that MMPs may be a “bridge” that mediates periodontitis to cause kidney disease still needs further confirmation.

### Influence if Oxidative Stress on Progression of CKD

Oxidative stress is defined as an imbalance between prooxidative and antioxidative systems and has been implicated in the development and/or progression of many inflammatory diseases, including PD. Oxidative stress is characterized by the accumulation of reactive oxygen species (ROS) (Golz et al., 2014). Moreover, oxidative stress caused by PD can also adversely affect the kidneys. Experimental periodontitis induced by simple ligature reportedly caused histomorphometric changes in renal tissues and damaged the brush border of renal tubules. Compared with animals without periodontitis, the malondialdehyde (MDA) content in the kidney of the periodontitis group was significantly increased, and glutathione (GSH) concentration was significantly reduced. To the best of our knowledge, this is the first study to demonstrate the development of significant renal histological changes and an increase in renal oxidative stress associated with periodontitis (Franca et al., 2017). However, in another study, antioxidants had protective effects on liver and kidney function damage caused by experimental PD. Melatonin, an antioxidant, has also been shown to decrease oxidative stress and periodontal inflammation in an experimental periodontitis rat model by downregulating inflammatory cytokines and restoring the antioxidant concentration (Kara et al., 2013). These studies suggest that oxidative stress plays an important role in the relationship between periodontitis and CKD.

Many studies have demonstrated the effects of periodontitis on circulating ROS and oxidative stress. Studies have shown that dental pulp collected from teeth with periodontitis is inflamed under oxidative stress and presents increased levels of apoptosis and autophagy relative to normal dental pulp (Li et al., 2019). The pathogenesis of periodontitis is related to an imbalance between ROS and the antioxidant defense system. For example, levels of thiobarbituric acid reactive substances such as MDA, a biomarker commonly employed for lipid peroxidation, are elevated in erythrocytes, plasma, and local tissue homogenates of periodontitis patients (Tsai et al., 2005; Cherian et al., 2019). MDA exists at greater levels in the GCF and saliva of patients with periodontitis compared to in healthy controls. In addition, the GCF concentrations of MDA/4-hydroxyalkanes were 200- to 400-fold greater than that in saliva, suggesting that the amount of ROS activity in GCF was much higher (Akalin et al., 2007). Periodontal inflammation leads to an increase in systemic proinflammatory cytokines, such as 8-Hydroxydeoxyguanosine (8-OHdG), dityrosine, nitric oxide metabolites (NOx), and nitrotyrosine. Improving 8-OHdG, dityrosine, NOx, nitrotyrosine, and proinflammatory cytokine levels has been shown to prevent ligature-induced periodontitis and alleviate systemic oxidative stress (Tamaki et al., 2014). In a mouse model of gingival sulcus, locally inducing periodontitis with LPS and proteases elevated gingival hexanoyl-lysine (HEL) expression, leading to elevated HEL levels in the serum and increased 8-OHdG levels in kidney tissue (Tomofuji et al., 2011). 8-OHdG is formed when guanine in DNA undergoes oxidative damage by ROS and lipid peroxide (Kasai, 2002). 8-OHdG is generally accepted as a reliable indicator of tissue oxidative damage (Tomofuji et al., 2006).

During periodontitis, ROS and inflammatory cytokines are released from immune cells to eliminate periodontal pathogens. However, these molecules are also thought to be important factors in the etiology of local tissue damage through the complex interactions between periodontal pathogenic bacteria and the host immune response (Chapple and Matthews, 2007). Periodontitis stimulates immune cells to produce ROS. Subgingival dental plaque is the main etiological agent for initiating inflammatory changes in the periodontal tissue and the cell component of bacteria, which exist in the dental plaque and recruit and activate hyper-responsive polymorphonuclear leukocytes (PMNs), which are innate immune cells that may play both defensive and destructive roles in periodontitis (Nicu and Loos, 2016) and accelerate oxidative stress biomarkers (Ademowo et al., 2020). Neutrophils produce ROS when responding to whole bacteria or their components. Some periodontitis pathogenic bacteria such as *A. actinomycetemcomitans*, *P. gingivalis*, and *Prevotella intermedia* LPS are potent inducers of neutrophil ROS production (Aida et al., 1995). Therefore, the immune response may be an important pathway for ROS to adversely affect the kidneys. In addition, tubular epithelial cell apoptosis was alleviated in LPS-induced acute kidney injury by decreasing ROS production (Golz et al., 2014). These results suggest that periodontitis can lead to renal tissue damage by increasing lipid peroxidation.

## Association Between PD and CKD

An increasing number of epidemiological and clinical data show that there is a correlation between periodontitis and CKD (Table 1), and the occurrence of periodontitis can have an impact on kidney tissue, function, and damage. Although animal experiments differ in design, the consequences of these experiments support this conclusion.

**TABLE 1:** Relationship between PD and CKD in clinical assessment.

Study authors/published time	Sample number	Research parameters	Type of study	Result	Conclusion
Han et al. (2013)	15,729 Korean adults from the Korean National Health and Nutritional Examination Surveys IV and V	eGFR, proteinuria, and hematuria	Cross-sectional study	1. PD patients: decreased eGFR, 4.07 (3.11–5.33); proteinuria, 2.12 (1.48–3.05); and hematuria, 1.25 (1.13–1.39, all *P* < 0.001) 2. Periodontitis was a significant predictor of decreased eGFR independent of all covariates [1.39 (1.03–1.89), *P* = 0.034] 3. Periodontitis was significantly correlated with hematuria [1.29 (1.15–1.46), *P* < 0.001]	The correlation between periodontitis and CKD markers Periodontitis was significantly correlated with hematuria.
Kim et al. (2017)	Low: 37 Moderate: 35 High: 35	PI, GI, PD, CAL	Cross-sectional study	1. Between groups— (χ^2^, *p* > 0.05) 2. Patients with severe periodontal disease: chance of high risk ↑ (odds ratio: 104.5; 95% CI: 10.7–1017.2; *p* < 0.0001).	1. Most of patients with chronic renal disease presented periodontal disease. 2. Patients with advanced periodontal disease had greater chance of high periodontal risk
de Souza et al. (2014)	CP: 73 (treated: 43; untreated: 30) No CP: 49	GI, PI, CI, PD, CAL, DMF-T, CRP, sCa, sP, sAP, serum albumin	Cohort study	1. CP vs. no CP: risk of death ↑ (hazard ratio 2.65 [95% confidence interval 1.06 to 6.59]; *P* = 0.036) 2. CP vs. a lesser extent for treated: (2.36 [1.01 to 5.59]; *P* = 0.047)	1. Poor oral health, including CP, is a common finding in patients undergoing HD. 2. CP patients had a higher risk of death.
Zhao et al. (2014)	HD patients: 102 Trauma patients: 204	AL, CPI, BL	Case control study	1. The CPI and AL showed statistical differences (*P* < 0.001). 2. BL: HD ↑ (*P* < 0.01), except the disto-buccal one (*P* < 0.05). 3. Furcation defect: HD was nearly double that of the controls (*P* < 0.001)	1. Periodontitis and periodontal BL were significantly more severe in the Chinese patients undergoing HD. 2. Periodontal disease may affect bone loss parameters in HD patients.
Sharma et al. (2016)	CKD: 861 Non-CKD: 12923	eGFR, HDL, CAL, total serum cholesterol, BOP, PPD, C-PPD	Cohort study	The 10-year all-cause mortality rate for individuals with CKD increased from 32% (95% CI: 29–35%) to 41% (36–47%) with the addition of periodontitis	Periodontitis is associated with mortality in patients with CKD
Kshirsagar et al. (2009)	Mild or no periodontal Disease: 100 Moderate-to-severe Disease: 68	Serum albumin, CRP, sCa, sP, sPTH, serum total cholesterol, serum ferritin	Retrospective cohort study	1. Moderate-to-severe disease was significantly associated with death from CVD causes, HR (95% confidence interval) 5.3 (1.5–18.9), *P* = 0.01 2. Adjustment for a variety of co-variables did not diminish the strength of this association. HR 5.0 (95% confidence interval, 1.2–19.1; *P* = 0.02).	Periodontitis could affect the progression of CVD.
Almeida et al. (2017)	CKD and severe CP: 26	PI, BOP, CAL, PPD, eGFR, triglycerides, total cholesterol, albumin and ADMA levels	Clinical trial	The median values (25%; 75% percentiles) of eGFR improve from 34.6 (27; 44.7) mL/min/1.73 m^2^ on baseline to 37.6 (29.7; 57) mL/min/1.73 m^2^ on day 90, and to 37.6 (28.6; 56) mL/min/1.73 m^2^ (*p* < 0.05) on day 180	CKD is associated with periodontitis. Periodontal treatment may be beneficial to the course of CKD.
Artese et al. (2010)	Predialysis patients: 21 CP without clinical evidence of kidney disease: 19	GB, VP, SUP, BOP, PD, AL, GFR, creatinine, calculus index	Clinical trial	Periodontal treatment had a statistically significant positive effect on the glomerular filtration rate of each individual (group 1, *p* = 0.04; group 2, *p* = 0.002)	Periodontal parameters improved after treatment. CKD predialysis patients show a good response to non-surgical periodontal treatment.
Graziani et al. (2010)	GCP systemically healthy subjects: 20	GFR, CRP, SAA, D-dimer, fibrinogen	Clinical trial	1. Cystatin C: ↓ from baseline to the end of the trial (*p* < 0.01). 2. CRP and SAA: ↑ (*p* < 0.001 versus baseline), while D-dimer (*p* < 0.05) and fibrinogen (*p* < 0.01) showed mild variations.	GFR may be positively affected by PT.
Lee et al. (2014)	Treatment: 35456 No treatment: 141824	Sex, age, and comorbidities	Retrospective cohort study	The incidence of ESRD in the treatment cohort: ↓ (4.66 versus 7.38 per 10,000 person-years), with an adjusted HR of 0.59 (95% CI = 0.46 to 0.75)	Surgical periodontal treatment reduced ESRD risk
Oyetola et al. (2015)	CRF and ESRD: 90 Controls: 90	GFR	Case control study	1. Oral lesions were present in 86 out of 90 (96.5%) CKD patients compared with 15 out of 90 (16.7%) controls (*p* < 0.001). 2. The mean GFR in subjects with oral lesions: ↓ *p* < 0.001.	1. Oral lesions are much more common in CKD patients. 2. The presence of oral lesions was positively associated with a decrease in GFR.
Honarmand et al. (2017)	HD: 30 Controls: 30	Saliva urea and calcium levels and pH values	Cross-sectional study	The mean salivary urea level (*p* = 0.0001) and pH value (*p* = 0.042) in the patient group: ↑	CKD patients display increased serum urea concentrations and elevated pH in the oral cavity

*GI, gingival index; PI, plaque index; PPD, probing pocket depth; BOP, bleeding on probing; CAL, clinical attachment level; DMF-T, decayed, missing, and filled teeth index; AL, attachment loss; CRP, C-reactive protein; PD, probing depth; CI, calculus index; sCa, serum calcium; sP, serum phosphorus; sAP, serum alkaline phosphatase; BL, bone loss; CPI, community periodontal index; C-PPD, cumulative periodontal probing depth; PPD, periodontal probing depth; eGFR, estimated glomerular filtration rate; HDL, high-density lipoprotein; sPTH, serum parathyroid hormone; ADMA, asymmetric dimethylarginine; VP, visible plaque index; GB, gingival bleeding index; SUP, suppuration; SAA, serum amyloid A; GFR, glomerular filtration rate; HD, hemodialysis; GCP, generalized chronic periodontitis; CP, chronic periodontitis; CKD, chronic kidney disease; CRF, chronic renal failure; ESRD, end stage renal disease; CVD, cardiovascular disease.*

Franca et al. (2017) established a Wistar rat model of periodontitis induced by simple ligation of the first mandibular molars. They performed hematoxylin and eosin (HE) staining, silver impregnation, and periodic acid–Schiff (PAS) staining to evaluate the brush edges of kidney tissue and histopathologically determined histomorphometric measurements (Franca et al., 2017). The results showed that in the periodontitis group, the renal cortex had obvious morphological changes, the renal tubular brush edge was damaged, and the renal tissue morphological measurements were significantly different from those in the control. It can be assumed that the development and progression of periodontitis can lead to changes in the form of renal tissue. In another study, three groups of male Wistar rats had their upper molars ligated for 3 weeks to induce periodontitis (Kose et al., 2020). One group of rats was treated with melinjo resveratrol, one without resveratrol, and the other was a control group with no periodontitis or resveratrol treatment. After inducing periodontitis, the rats were provided with water with or without melinjo resveratrol. The results showed that drug therapy could improve the local condition of gingival redox balance under periodontitis conditions and reduce the circulating oxidative stress; meanwhile, a decrease in the level of oxidative stress could alleviate kidney injury. Therefore, periodontitis can increase the level of circulating oxidative stress, thus causing kidney damage.

Furthermore, periodontitis induced by periodontal pathogens can have an influence on and damage the kidneys. Harada et al. (2018) studied the effect of gingival polysaccharide (PG-LPS) on the kidneys of mice. HE and PAS staining were performed on the kidneys for morphological and immunohistochemical analyses (Harada et al., 2018). The glomeruli were immunopositive for eNOS in the experimental group, but not in the control group. However, the glomerular capillaries and tubules appeared normal in both the HE and PAS staining groups. In another mouse model, local periodontitis was induced by introducing LPS into the gingival sulcus of the maxillary first molar. In this model, no renal tissue damage or pathological changes were observed in either the periodontitis group or the control group, but periodontitis could cause kidney damage by increasing lipid peroxidation (Tomofuji et al., 2011). In one study, when pathogenic bacteria were introduced into the GCF to construct a mouse model of periodontitis, the serum levels of AST, ALT, and BUN were significantly higher in the LPS group than in the control group (Gulle et al., 2014). After LPS stimulation, periodontitis usually results in hepatic and renal dysfunction. Serum aspartate aminotransferase (AST), alanine transaminase (ALT), and blood urea nitrogen (BUN) levels were elevated after periodontitis was improved with drugs. These results show that experimental treatment of periodontitis can alleviate kidney disease, thus demonstrating the damage to the kidneys caused by periodontitis.

Moreover, periodontitis can cause changes in the kidneys as a result of complications of obesity or diabetes. Chen et al. (2017) studied the probable mechanism and impact of periodontitis on kidney injury in obese mice. Their results showed that periodontitis exacerbated pathological changes in the kidneys of obese mice on a high-fat diet. The specific mechanism by which this occurs may involve downregulation of MMP2 and upregulation of MMP inhibitors, tissue inhibitors of metalloproteinases-1 (TIMP1), and TGF-β1. Furthermore, lean mice with periodontitis were found to have increased glucose tolerance compared to mice without periodontitis (Pontes Andersen et al., 2007). The average concentration of IL-1β was also increased in Zucker fatty rats (ZFRs) compared with in those without periodontitis. The mean concentration of IL-1 in ZFRs with periodontitis was higher than that in lean mice without periodontitis, and significantly higher than that in healthy lean mice. Periodontitis is associated with impaired glucose metabolism in ZFRs, indicating that periodontitis is a contributing factor to diabetes. Disordered sugar homeostasis is also related to the presence of periodontitis in lean rats. The first study evaluating the development of early periodontitis-associated renal changes in ZFRs found that hypertrophy of the kidney, increased glomerular volume, and cardiac weight in prediabetic patients were associated with periodontitis (Pontes Andersen et al., 2008). In ZFRs without prediabetes, the mRNA levels of fibronectin were elevated during periodontitis. Ligated periodontitis is associated with an increased glomerular volume and weight. Fibronectin expression was increased in emaciated rats with periodontitis. This suggests that periodontitis affects early renal changes in diabetes mellitus. Another study found that the relative accumulation of type 1 collagen in the glomeruli of streptozotocin (STZ)-induced type 1 diabetic mice repeatedly administered *P. gingivalis* LPS was higher than that in mice not administered LPS (Sawa et al., 2014). In addition, more cytokines associated with glomerular sclerosis were produced in the glomeruli of diabetic mice that were repeatedly treated with LPS than in the glomeruli of untreated diabetic mice or LPS-administered non-diabetic mice. Overall, data from these experimental models illustrate the association between periodontal pathogens and renal disease.

It has been reported that oral lesions are more common in patients with CKD, while serum urea concentrations increase and oral pH increases in these patients (Oyetola et al., 2015; Honarmand et al., 2017). The results (Oyetola et al., 2015) showed that the mean GFR of patients with oral lesions was significantly lower than that of patients without oral lesions (*P* < 0.001). This finding suggests a strong relationship between the presence of oral lesions and the reduction of GFR in patients with CKD, suggesting a possible role of oral lesions in the initiation and/or progression of renal disease. Some reports have highlighted the increased incidence of PD in patients with CKD, and oral changes may predispose patients with CKD to PD (Schutz et al., 2020). In a subsequent cross-sectional study of 15,729 Korean adults, Han et al. (2013) found that the risk of hematuria in patients with periodontitis and CKD was 1.25 higher than that in patients without periodontitis. In two independent models created by Fisher et al. (2011), PD (adjusted odds ratio 1.62), edentulism (adjusted odds ratio 1.83), and the PD score were associated with CKD when simultaneously adjusting for 14 other factors. The final results showed that there was a bidirectional relationship between PD and CKD, demonstrating that both were risk factors for each other.

In recent years, an increasing number of studies have confirmed the close relationship between PD and dialysis, including peritoneal dialysis and HD, and clarified the relationship between CKD and PD. Peritoneal dialysis or HD treatment stratifies the prevalence and severity of PD. The observation rate of patients on continuous ambulatory peritoneal dialysis (CAPD) was as high as 42.6% (Kocyigit et al., 2014). Surprisingly, a recent report demonstrated that 107 HD patients (99.1%) showed some form of periodontitis (Kim et al., 2017), and another study showed that only one in 103 HD patients had healthy periodontal tissue (Cholewa et al., 2018). Similarly, de Souza et al. (2014) showed that symptoms of poor oral health, including chronic periodontitis, are often found in patients with HD. In Chinese patients, BL was significantly higher in patients with HD at their mandibular first premolars and first molars than in patients without HD (*P* < 0.01) at every site (except the disto-buccal site) (*P* < 0.05) (Zhao et al., 2014). Moreover, PD may also affect nutritional and bone loss parameters in HD patients (Zhao et al., 2014; Cholewa et al., 2018).

Statistically, there is a strong correlation between periodontitis and increased mortality in patients with CKD. CKD patients have an extraordinarily high risk of death, and chronic inflammation is increasingly recognized as a risk factor. Furthermore, periodontitis is one of the sources of systemic inflammation in patients with CKD (Yazdi et al., 2013; de Souza et al., 2014). When the 10-year survival of the most recent group of patients with CKD was analyzed, the mortality rate of patients increased from 32% to 41% when periodontitis was present (Sharma et al., 2016). A meta-analysis of a cohort study confirmed that PD was associated with an increased risk of death from CKD (Zhang et al., 2017). The results of the research by de Souza et al. (2014) showed that in a univariate analysis of untreated patients, patients with chronic periodontitis had a higher risk of death from all causes than patients without PD (hazard ratio 2.65, 95% CI: 1.06–6.59, *P* = 0.036) and treated patients (hazard ratio 2.36, 95% CI: 1.01–5.59, *P* = 0.047) (de Souza et al., 2014). In fact, PD-induced systemic inflammation was identified as a potential cause and/or non-traditional risk factor for CKD progression (Wahid et al., 2013). In addition, periodontitis can affect the progression of cardiovascular disease (CVD) (Kshirsagar et al., 2009) and markedly increase the risk of CVD-related mortality in CKD patients (de Souza et al., 2014).

Among the included studies, renal function was assessed using at least one of the following parameters: eGFR, BUN, and serum creatinine levels. The periodontal status can be assessed using various parameters. After non-surgical periodontal treatment (NSPT), almost all periodontal parameters in patients with CKD were significantly improved, demonstrating its effectiveness.

In two case series studies, eGFR was measured at baseline and follow-up. Both studies reported a statistically significant increase in eGFR among CKD patients (stages 2–4) (Artese et al., 2010; Almeida et al., 2017). Studies have shown that patients with CKD before dialysis have a higher prevalence of inflammation (Stenvinkel et al., 2000). The findings showed that periodontal therapy was beneficial to endothelial and renal function, possibly by reducing inflammation, which may indicate a new therapeutic approach in this population (Almeida et al., 2017). In addition, among systematically healthy subjects, urea, creatinine, and GFR were not significantly changed before or after NSPT, while cystatin C significantly decreased after 1-, 3-, and 6-month follow-up compared with the baseline value (Graziani et al., 2010). Lee et al. (2014) concluded that surgical periodontal treatment reduced the risk of end-stage renal disease in a retrospective cohort study based on insurance claims data. This suggests that closer collaboration between doctors and dentists may facilitate the treatment of patients with CKD. Although periodontitis is a common disease, it can be prevented and cured in most cases. However, periodontitis is often perceived as relatively harmless compared with other life-threatening diseases. It is common for patients with severe CKD to neglect their oral hygiene, even when they present with periodontitis. Although clinical data confirm an increased prevalence of periodontitis in patients with CKD, self-reported awareness of periodontitis in patients is low (Dannewitz et al., 2020). Tooth and gum loss may accelerate in CKD, and patients with CKD are less likely to receive dental care due to poor oral hygiene (Grubbs et al., 2012). However, there are also studies suggesting that patients with CKD who have not started dialysis have a poor response to periodontal treatment because of their weakened immune function (Cohen et al., 1997; Vanholder et al., 2003).

## Author Contributions

B-ZL and TX participated in the review selection and design. LL, Y-LZ, and X-YL was in charge of the writing of the review. XM was responsible for figures and tables. R-QZ and L-LO was involved in the revision of the language. All authors contributed to the article and approved the submitted version.

## Conflict of Interest

The authors declare that the research was conducted in the absence of any commercial or financial relationships that could be construed as a potential conflict of interest.
